# Lung function in Birt-Hogg-Dubé syndrome: a retrospective analysis of 96 patients

**DOI:** 10.1186/s13023-020-01402-y

**Published:** 2020-05-24

**Authors:** C. Daccord, V. Cottin, G. Prévot, Y. Uzunhan, J. F. Mornex, P. Bonniaud, R. Borie, A. Briault, M. A. Collonge-Rame, B. Crestani, G. Devouassoux, O. Freynet, A. Gondouin, P. A. Hauss, C. Khouatra, S. Leroy, S. Marchand-Adam, C. Marquette, D. Montani, J. M. Naccache, G. Nadeau, N. Poulalhon, M. Reynaud-Gaubert, M. Salaun, B. Wallaert, J. F. Cordier, M. Faouzi, R. Lazor, the OrphaLung network

**Affiliations:** 1 Service de pneumologie, Centre hospitalier universitaire vaudois, Université de Lausanne, Rue du Bugnon 46, CH-1011 Lausanne, Switzerland; 2 Service de pneumologie, Centre national coordinateur de référence des maladies pulmonaires rares, hôpital Louis Pradel, Hospices Civils de Lyon, Université de Lyon, Université Claude Bernard Lyon 1, UMR754 INRA, IVPC, Lyon, France; 3 Service de pneumologie, Centre hospitalier universitaire de Toulouse, Toulouse, France; 4 Service de pneumologie, Assistance Publique Hôpitaux de Paris, Hôpital Avicenne, INSERM UMR 1272, Université Paris 13, Bobigny, France; 5 Service de Pneumologie et Soins Intensifs Respiratoires, Centre hospitalier universitaire Dijon/Bourgogne, Université Bourgogne-Franche Comté, INSERM U123-1, Dijon, France; 6 Service de pneumologie, Assistance Publique Hôpitaux de Paris, Hôpital Bichat - Claude Bernard, Paris, France; 7 Service de pneumologie, Centre hospitalier universitaire de Grenoble, Grenoble, France; 8 Service de génétique biologique - histologie, UF cytogénétique, UF consultations d’oncogénétique, Centre hospitalier universitaire de Besançon, Besançon, France; 9 Service de pneumologie, Hospices Civils de Lyon, Hôpital de la Croix-Rousse, Lyon, France; 10 Service de pneumologie, Centre hospitalier universitaire de Besançon, Besançon, France; 11 Centre hospitalier intercommunal Elbeuf - Louviers - Val de Reuil, Elbeuf, France; 12 Service de pneumologie, Université Côte d’Azur, Centre hospitalier universitaire de Nice, CNRS, INSERM, FHU OncoAge, Nice, France; 13 Service de pneumologie, Centre hospitalier universitaire de Tours, Tours, France; 14 Service de Pneumologie, Université Paris–Sud, Assistance Publique Hôpitaux de Paris, INSERM UMR S999, Hôpital de Bicêtre, Le Kremlin Bicêtre, France; 15 Service de Pneumologie, Site constitutif du Centre de référence des maladies pulmonaires rares OrphaLung, Assistance Publique Hôpitaux de Paris, Hôpital Tenon, Paris, France; 16 Centre hospitalier Métropole Savoie, UF de Génétique chromosomique, Chambéry, France; 17 Service de dermatologie, Hospices Civils de Lyon, Centre hospitalier Lyon-Sud, Lyon, France; 18 Service de pneumologie, Centre de compétences des maladies pulmonaires rares, Assistance Publique Hôpitaux de Marseille, Centre hospitalier universitaire de Marseille, Aix Marseille Université, Marseille, France; 19 Service de pneumologie, Centre hospitalier universitaire de Rouen, Rouen, France; 20 Service de pneumologie, Centre hospitalier universitaire de Lille, Lille, France; 21 Division de biostatistique, Centre universitaire de médecine générale et santé publique (Unisanté), Université de Lausanne, Lausanne, Switzerland

**Keywords:** Birt-Hogg-Dube syndrome, *FLCN* protein, human, Respiratory function tests, Pleurodesis

## Abstract

**Background:**

Birt-Hogg-Dubé syndrome (BHD) is a rare autosomal dominant disorder caused by mutations in the *FLCN* gene coding for folliculin. Its clinical expression includes cutaneous fibrofolliculomas, renal tumors, multiple pulmonary cysts, and recurrent spontaneous pneumothoraces. Data on lung function in BHD are scarce and it is not known whether lung function declines over time. We retrospectively assessed lung function at baseline and during follow-up in 96 patients with BHD.

**Results:**

Ninety-five percent of BHD patients had multiple pulmonary cysts on computed tomography and 59% had experienced at least one pneumothorax. Mean values of forced expiratory volume in 1 second (FEV_1_), forced vital capacity (FVC), FEV_1_/FVC ratio, and total lung capacity were normal at baseline. Mean (standard deviation) residual volume (RV) was moderately increased to 116 (36) %pred at baseline, and RV was elevated > 120%pred in 41% of cases. Mean (standard deviation) carbon monoxide transfer factor (DLco) was moderately decreased to 85 (18) %pred at baseline, and DLco was decreased < 80%pred in 33% of cases. When adjusted for age, gender, smoking and history of pleurodesis, lung function parameters did not significantly decline over a follow-up period of 6 years.

**Conclusions:**

Cystic lung disease in BHD does not affect respiratory function at baseline except for slightly increased RV and reduced DLco. No significant deterioration of lung function occurs in BHD over a follow-up period of 6 years.

## Introduction

Birt-Hogg-Dubé syndrome (BHD) is a rare inherited autosomal dominant disorder first described in 1977 [1], and caused by mutations in the tumour suppressor gene *FLCN* coding for folliculin [2]. Its clinical expression includes cutaneous fibrofolliculomas, renal tumours of various histological types, and multiple pulmonary cysts. The condition exhibits a wide phenotypic variability. Affected individuals can present with any combination of skin, pulmonary, or renal manifestations of varying degrees of severity, even within the same family.

More than 80% of patients with BHD present with multiple bilateral pulmonary cysts on high-resolution computed tomography (HRCT) [3–5]. The cysts can vary in size, shape and number, but are typically thin-walled and predominantly distributed in the basal and subpleural or paramediastinal regions of the lung, with a normal-appearing surrounding parenchyma [6, 7]. The presence of cysts predisposes to spontaneous pneumothorax, with an incidence 50-fold higher than in the general population [3] and a high recurrence rate. Therefore, pleurodesis is recommended after the first episode of pneumothorax [8, 9].

Apart from episodes of pneumothorax, pulmonary cysts are usually asymptomatic, although mild exertional dyspnoea and/or cough have been occasionally reported [10]. Cystic lung disease in BHD has not been reported as leading to respiratory failure, even when extensive. This contrasts with lymphangioleiomyomatosis (LAM), another multiple cystic lung disease characterized by progressive destruction of the lung parenchyma, airflow obstruction, and accelerated lung function decline which may lead to respiratory insufficiency and require lung transplantation [11].

Until recently, data on lung function in BHD were limited to case reports or small series with baseline data only [10, 12–14], but whether pulmonary function declines during follow-up was unknown. A recent small Korean series observed a stability of forced vital capacity in 9 patients over a median period of 52 months [15]. However, lung function course has not been studied previously in a large series. The aims of this study were therefore: 1) to determine lung function at baseline and during follow-up in patients with BHD, 2) to determine whether lung function in BHD is correlated with age, gender, smoking history and previous pleurodesis.

## Materials and methods

### Study design and case selection

We performed a retrospective multicentric study on lung function data at baseline and over disease course in a large series of patients with BHD. The inclusion criteria were a diagnosis of BHD and at least one lung function test available. The diagnosis of BHD was established according to the criteria proposed by Menko et al. [16]. Cases were recruited through OrphaLung, a network of French physicians interested in rare lung diseases (formerly Groupe d’Études et de Recherche sur les Maladies Orphelines Pulmonaires, GERMOP). Participating centers included all cases seen up to data collection. From 99 cases available for analysis, 96 met the inclusion criteria. The remaining 3 cases were excluded due to diagnostic uncertainty in 2, and unavailable lung function in one.

### Data collection

Demographic, clinical, genetic and lung function data were collected from medical records through questionnaires filled by the referring physicians. Anonymised data were sent to the investigators. Only lung function parameters measured in stable condition were used. Forced expiratory volume in one second (FEV_1_) and forced vital capacity (FVC) were measured by spirometry. Total lung capacity (TLC) and residual volume (RV) were measured by body plethysmography [17, 18]. Carbon monoxide transfer factor (DLco) and coefficient (DLco/V_A_) were assessed with the single-breath method [19]. Lung function parameters were expressed in percentage of predicted values (%pred) using the 1993 European Community of Coal and Steel reference equations [20]. FEV_1_/FVC and RV/TLC ratios were expressed as absolute percentage (%). Arterial oxygen partial pressure (PaO_2_) was expressed in mmHg. Lung function test results were interpreted according to the American Thoracic Society/European Respiratory Society guidelines [21]. For each lung function measurement in each patient, a history of previous unilateral or bilateral pleurodesis was recorded.

### Data analysis

Data were expressed as proportions for categorical variables, and by mean and standard deviation (SD) for continuous variables. Outcomes were log transformed when the normality assumption was violated. As time periods between consecutive lung function tests varied, dates of measurements were rounded to 1-year intervals. If 2 or more measurements were performed during a given 1-year interval, mean values over this year were used. Follow-up duration was limited to 6 years, as the number of cases with longer follow-up was very low (*n* = 6).

Associations between respiratory function outcomes at baseline and respectively age, gender, smoking, and history of pleurodesis were analysed with linear robust regression [22, 23]. Associations between the same variables and respiratory function during follow-up were analysed with a linear mixed model. Univariate and adjusted analyses were performed. As full details on genetic mutations were available in only two thirds of cases, no attempt was made to identify genotype-phenotype associations. Statistical analyses were performed with Stata 14 software (StataCorp. 2015. Stata Statistical Software: Release 14. College Station, TX: StataCorp LP). A *p*-value < 0.05 was considered significant.

## Results

### Patient characteristics

Demographic and clinical features of the 96 patients (75 families) with BHD are presented in Table 1. A pathogenic *FLCN* gene mutation was identified in 89 patients (93%). In the other 7 patients (7%), the diagnosis was based on a combination of clinical and imaging diagnostic criteria. The mean (SD) age at diagnosis was 48 (14) years, and 52% were males. Fifty-one percent were current or ex-smokers, with a mean (SD) consumption of 14 (10) pack-years. Pulmonary cysts were found on HRCT in 95% of cases. Seventy-nine percent presented with cutaneous manifestations, and 11% had renal tumours. Sixty-one percent of patients had a family history of pneumothorax and/or pulmonary cysts. Mild to moderate dyspnea and cough were reported in a minority of cases. Chest pain outside episodes of pneumothorax and hemoptysis were uncommon. Fifty-nine percent had experienced at least one pneumothorax. Forty-four percent of patients had undergone at least one pleurodesis (unilateral in 30% and bilateral in 14%).

**Table 1:** Patient characteristics in 96 cases of BHD

	Cases with available data, n	value
Diagnosis by *FLCN* gene mutation, %	96	93
Diagnosis by combination of other criteria, %	96	7
Age at diagnosis, mean (SD)	96	48 (14)
*Age at diagnosis in men, mean (SD)*	*50*	*49 (15)*
*Age at diagnosis in women, mean (SD)*	*46*	*48 (14)*
Male sex, %	96	52
Never-smokers, %	95	49
*Never-smokers in men, %*	*50*	*40*
*Never-smokers in women, %*	*45*	*60*
Active smokers, %	95	13
*Active smokers in men, %*	*50*	*16*
*Active smokers in women, %*	*45*	*9*
Former smokers, %	95	38
*Former smokers in men, %*	*50*	*44*
*Former smokers in women, %*	*45*	*31*
Pack-years, mean (SD)	47	14 (10)
Pulmonary cysts on HRCT, %	93	95
Cutaneous manifestations, %	89	79
Renal tumours, %	95	11
Familial history of pneumothorax and/or pulmonary cysts, %	77	61
Familial history of BHD, %	60	60
Dyspnea, %	93	34
Cough, %	93	15
Chest pain, %	88	5
Hemoptysis, %	91	1
Pneumothorax (≥ 1), %	96	59
Age at first pneumothorax, mean (SD)	15	33 (13)
Number of pneumothoraces, mean (SD)	96	1.6 (2.3)
Pleurodesis, none / unilateral / bilateral, %	94	56 / 30 / 14

### Lung function at baseline

Lung function parameters at baseline were normal in the vast majority of cases, with mean lung volumes, FEV_1_/FVC, DLco/V_A_ and PaO_2_ values within the normal range (Table 2). The main abnormalities were a slightly increased RV to a mean (SD) value of 116 (36) %pred, and a slightly reduced DLco to a mean (SD) value of 85 (18) %pred. Only 14% of patients had FEV_1_/FVC ratio < 70, 41% had RV values > 120%pred, and 33% had DLco values < 80%pred.

**Table 2:** Lung function parameters at baseline

	cases with available data, n	mean (SD)	abnormal values criterion	frequency of abnormal values, %
FEV_1_, %pred	96	97 (16)	< 80	8
FVC, %pred	96	102 (16)	< 80	6
FEV_1_/FVC, %	94	78 (7)	< 70	14
TLC, %pred	70	103 (14)	< 80 / > 120	3 / 9
RV, %pred	69	116 (36)	> 120	41
RV/TLC, %	60	36 (9)	> 40	15
DLco, %pred	69	85 (18)	< 80	33
DLco/V_A_, %pred	70	94 (19)	< 80	24
PaO_2_, mmHg	20	90 (9)	< 75	5

*FEV*_*1*_ forced expiratory volume in one second, *FVC* forced vital capacity, *TLC* total lung capacity, *RV* residual volume, *DLco* carbon monoxide transfer factor, *DLco/V*_*A*_ carbon monoxide transfer coefficient, *PaO*_*2*_ arterial oxygen partial pressure, *%pred* percentage of predicted value

Associations between lung function parameters at baseline and respectively age, gender, smoking history, and previous unilateral or bilateral pleurodesis are shown in Table 3. When adjusted for gender, age and smoking, bilateral pleurodesis was associated with significantly lower FEV_1_ (β = − 13.40; *p* = 0.003), lower FVC (β = − 20.74; *p* < 0.001), and higher FEV_1_/FVC (β = 5.52; *p* = 0.024). Unexpectedly, female sex was associated with significantly higher RV (β = 19.89; *p* = 0.03) and RV/TLC (β = 6.84; p = 0.003), lower DLco (β = − 13.58; *p* = 0.002), and lower DLco/V_A_ (β = − 18.50; *p* < 0.001). Smoking was associated with lower DLco (β = − 8.93; *p* = 0.033). PaO_2_ was not associated with any of the variables examined (data not shown).

**Table 3:** Multivariable robust regression analysis of the associations between clinical characteristics and baseline lung function

Variable	FEV_**1**_ (%pred)		FVC (%pred)		FEV_**1**_/FVC (%pred)		TLC (%pred)		RV (%pred)		RV/TLC (%)		DLco (%pred)		DLco/V_**A**_ (%pred)	
	β	p	β	p	β	p	β	p	β	p	β	p	β	p	β	p
**Age (yr)**	0.19	0.062	0.20	0.058	−0.09	0.129	0.07	0.591	−0.30	0.320	0.22	**0.007**	−0.17	0.259	0.03	0.848
**Gender (F)**	−2.31	0.438	2.34	0.444	−0.84	0.610	7.18	0.057	19.89	**0.030**	6.84	**0.003**	−13.58	**0.002**	−18.50	**< 0.001**
**Smoking (yes)**	0.24	0.935	2.55	0.395	−1.95	0.226	1.63	0.654	3.75	0.669	−0.86	0.687	−8.93	**0.033**	−9.11	0.053
**Pleurodesis**																
**Unilateral**	−5.70	0.090	−6.22	0.071	−0.19	0.918	−0.72	0.860	10.22	0.301	4.38	0.083	2.73	0.558	5.10	0.339
**Bilateral**	−13.40	**0.003**	−20.74	**< 0.001**	5.52	**0.024**	−4.26	0.432	25.51	0.055	11.07	**0.001**	−9.51	0.141	4.21	0.541

*FEV*_*1*_ forced expiratory volume in one second, *FVC* forced vital capacity, *TLC* total lung capacity, *RV* residual volume, *DLco* carbon monoxide transfer factor, *DLco/V*_*A*_ carbon monoxide transfer coefficient, *yr* year, *F* female, *%pred* percentage of predicted value, *β* partial regression coefficient. Robust linear regression model

### Lung function during follow-up

**Fig. 1:**
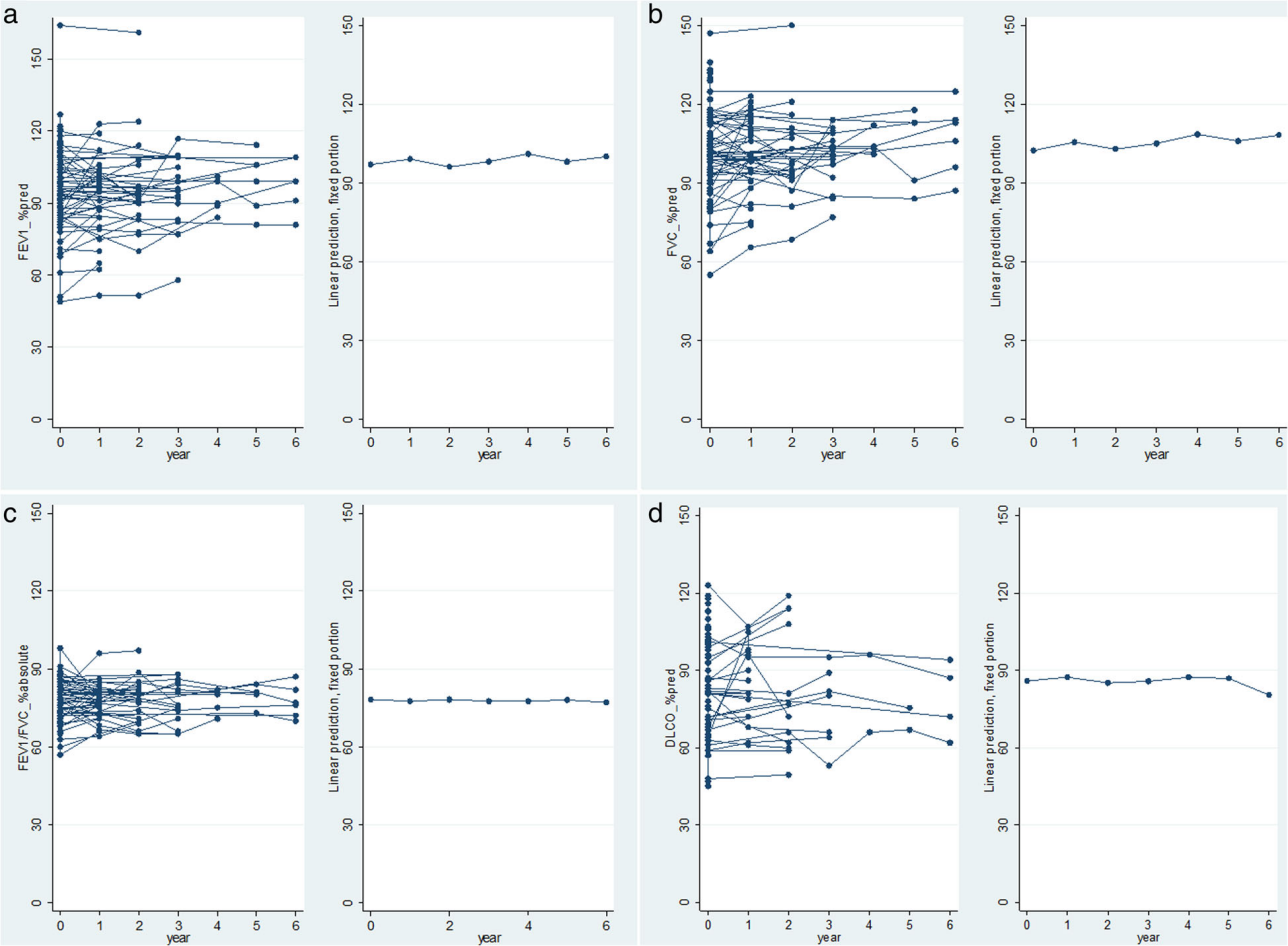
Evolution of lung function parameters during follow-up in BHD for **a** FEV_1_, **b** FVC, **c** FEV_1_/FVC, **d** DLco. Left: raw data, right: linear prediction fixed proportion model

Follow-up data were available in 57 patients. The mean follow-up duration was 2.8 (3.5) years, and the mean number of visits was 3.1 (1.7). FEV_1_, FVC, FEV_1_/FVC, TLC, RV, RV/TLC, DLco, DLco/V_A_, and PaO_2_ were not significantly and consistently different from baseline over a follow-up period of up to 6 years (Table 4). The course of FEV_1_, FVC, FEV_1_/FVC and DLco is illustrated in Fig. 1.

**Table 4:** Multivariable linear mixed model of the associations between clinical characteristics and lung function during follow-up

Variable	FEV_1_ (%pred)		FVC (%pred)		TLC (%pred)		RV (%pred)		RV/TLC (%)		DLco (%pred)		DLco/V_A_ (%pred)	
	β	p	β	p	β	p	β	p	β	p	β	p	β	p
**Time**														
**year 1**	2.23	0.122	3.22	**0.045**	1.45	0.511	−5.80	0.402	−1.59	0.265	1.31	0.629	−2.04	0.421
**year 2**	−1.15	0.476	0.14	0.937	−0.47	0.862	−12.95	0.111	−3.18	0.055	−0.97	0.745	−4.15	0.127
**year 3**	0.92	0.598	2.37	0.234	5.97	**0.043**	−10.7	0.910	− 1.43	0.451	−0.56	0.867	1.97	0.542
**year 4**	5.28	0.095	7.41	**0.035**	−0.64	0.936	−31.4	0.233	−1.97	0.703	−0.04	0.994	^a^	^a^
**year 5**	1.83	0.564	4.41	0.212	6.59	0.263	1.57	0.934	1.71	0.650	0.87	0.887	−0.31	0.967
**year 6**	3.88	0.198	6.68	**0.045**	5.71	0.187	−4.74	0.738	0.65	0.824	−5.76	0.242	−10.5	0.121
**Age (yr)**	0.24	**0.010**	0.19	**0.035**	0.04	0.667	−0.31	0.894	0.24	**< 0.001**	− 0.14	0.248	0.04	0.759
**Gender (F)**	−6.54	**0.024**	−0.80	0.768	5.62	0.051	20.61	**0.001**	7.74	**< 0.001**	−14.26	**< 0.001**	−14.0	**< 0.001**
**Smoking (yes)**	−1.05	0.712	1.49	0.580	1.60	0.575	−0.84	0.894	−0.77	0.607	−7.31	**0.044**	−7.41	0.064
**Pleurodesis**														
**Unilateral**	−4.02	0.165	−5.32	0.059	−0.59	0.859	5.82	0.422	3.24	0.068	0.32	0.938	4.27	0.354
**Bilateral**	−12.96	**< 0.001**	−14.72	**< 0.001**	−4.68	0.249	15.82	0.068	7.98	**< 0.001**	0.96	0.834	6.29	0.263

*FEV*_*1*_ forced expiratory volume in one second, *FVC* forced vital capacity, *TLC* total lung capacity, *RV* residual volume, *DLco* carbon monoxide transfer factor, *DLco/V*_*A*_ carbon monoxide transfer coefficient, *yr* year, *F* female, *%pred* percentage of predicted value, *β* partial regression coefficient. Linear mixed model. ^a^omitted due to missing data

Multivariable associations between lung function course during follow-up and age, gender, smoking, and unilateral or bilateral pleurodesis are shown in Table 4. FEV_1_ (β = 0.24; *p* = 0.01), FVC (β = 0.19; *p* = 0.035), and RV/TLC (β = 0.24, p < 0.001) significantly increased with age. Female sex was associated with lower FEV_1_ (β = − 6.54; p = 0.024), higher RV (β = 20.61; *p* = 0.001) and RV/TLC (β = 7.74; p < 0.001), and lower DLco (β = − 14.26; p < 0.001) and DLco/V_A_ (β = − 14.0, p < 0.001). Smoking was only associated with lower DLco (β = − 7.31; *p* = 0.044). As seen at baseline, bilateral pleurodesis was associated with lower FEV_1_ (β = − 12.96; p < 0.001), lower FVC (β = − 14.72; p < 0.001), and higher RV/TLC (β = 7.98, p < 0.001) but not with other parameters. FEV_1_/FVC ratio was not significantly associated with any variable (data not shown). Owing to small number of available data, PaO_2_ was excluded from these analyses.

## Discussion

To our knowledge, this is the first large study assessing lung function at baseline and during follow-up in BHD. The main findings are that lung function tests and PaO_2_ were normal in most patients at baseline except for increased RV and reduced DLco in a minority, and that no deterioration occurred over a follow-up period of 6 years. These findings sharply contrast with the natural history of LAM, which is usually associated with reduced FEV_1_ and DLco at baseline, and accelerated lung function decline over time.

The most common lung function abnormality at baseline in our patients was increased RV, found in 41% of cases, with a mean (SD) value for the whole study population of 116 (36) %pred (Table 2). This abnormality could be attributed to the space-occupying effect of pulmonary cysts. Increased RV is also a feature of LAM [24–26], in which it appears more pronounced, probably as a result of the more severe involvement of the lung parenchyma. The second most common finding in the present series was reduced DLco, observed in 33% of cases, with a mean (SD) value for the whole study population of 85 (18) %pred (Table 2). It can be attributed to the loss of alveolar units available for gas exchange secondary to cystic destruction of the lung parenchyma, and also possibly to ventilation-perfusion inequality. Indeed, in one quantitative analysis of the lung parenchyma by HRCT in patients with BHD, the volume occupied by cysts in the whole lung was on average 13% [6]. In another study of patients with BHD, the proportion of low attenuation areas (i.e. cysts) at HRCT was 5.2% in the upper lung zones, 4.2% in the middle lung zones and 9.9% in the lower lung zones [27]. Thus, the loss of lung parenchyma induced by pulmonary cysts may explain a significant part of the 15% loss of DLco observed in our study. Additionally, smoking was associated with DLco decrease in our population (Table 3), and contributed to reduce mean DLco, probably through the occurrence of subclinical emphysema in smokers. Reduced DLco is also common in LAM [28–30] and appears more severe in this disorder. An obstructive ventilatory defect was uncommon in our series (14% of cases), whereas it is a frequent feature of LAM.

We found that FEV_1_ and FVC slightly but significantly increased with age during follow-up. Similarly, RV/TLC also significantly increased with age, whereas RV did not. These changes are unexpected, as a decrease of FEV_1_ and FVC over time is usually observed in multiple cystic lung diseases. We do not have an explanation for these findings. An increase of RV/TLC could have occurred as a result of increasing cyst volume over time, but this could not be assessed in this study.

Female patients had significantly higher RV and RV/TLC both at baseline and during follow-up, as well as significantly lower FEV_1_ during follow-up. They also had lower DLco and DLco/V_A_ than males at baseline and during follow-up, after adjustment for other variables. Since DLco and DLco/V_A_ measurements were not corrected for hemoglobin level in this study, a bias cannot be ruled out, although anemia is not a feature of BHD, and the lower hemoglobin level in the female population is taken into account in DLco prediction equations. However, to explain the difference between men and women observed in this study, one could also hypothesize that women with BHD truly have a more severe lung involvement due to hormonal factors. Estrogens likely modulate disease course in LAM, a disorder which almost exclusively affects women, may be worsened by pregnancy [28, 31], and becomes milder after menopause [32]. This hormonal modulation in LAM might result from interactions between the estrogen signalling pathway and the mechanistic target of rapamycin (mTOR) [33], a key player in the pathogenesis of LAM. As folliculin appears involved in the regulation of cell growth, proliferation and survival through interactions with mTOR [34, 35], a similar modulatory role of estrogens cannot be ruled out in BHD. However, BHD affects equally women and men [8], and two large series showed no gender predilection for the prevalence of pulmonary cysts and pneumothorax in BHD [3, 5]. Whether men and women truly have a different disease severity in BHD deserves further study.

As expected, smokers had lower DLco at baseline and during follow-up, but smoking was not significantly associated with other lung function parameters. This could be explained by the relatively short follow-up period, and a relatively young study population (mean age 48 years) as compared to the average age of onset of chronic obstructive pulmonary disease, as well as a relatively low mean cumulative tobacco consumption (14 pack-years).

Patients who underwent bilateral pleurodesis had significantly lower FEV_1_ and FVC at baseline and during follow-up, and significantly higher FEV_1_/FVC ratio at baseline, as compared to patients without pleurodesis. Patients with unilateral procedure tended to have similar changes, although not reaching statistical significance. These findings are expected, and reflect the restrictive effect of the procedure, especially when bilateral.

With respect to lung function course over time, our data demonstrate that no deterioration occurs in BHD even after a prolonged observation period of up to 6 years, and that BHD thus behaves differently from LAM. Consequently, lung function follow-up may not be mandatory in BHD, in contrast to LAM where a regular follow-up is recommended [11].

This study has several limitations. First, its retrospective nature and the relatively small sample size compel us to interpret our observations with caution, although participating centers included all consecutive cases seen in the study period. Secondly, one could argue that cases were mainly recruited by respiratory physicians, and this may have selected cases with more frequent pulmonary involvement. However, as most cases were seen at university hospitals, a proportion was primarily diagnosed by dermatologists, nephrologists or geneticists, and only secondarily referred to respiratory physicians, thus minimizing this potential source of bias. In addition, the prevalence of pulmonary cysts and pneumothorax was similar to previous series. Thirdly, the multicentric nature of the study is a source of variability in pulmonary function test measurements. However, each patient was followed over time at the same center and was his own comparator during follow-up, which has limited this potential cause of bias. Another limitation is that our search of variables associated with lung function was restricted to age, gender, smoking and pleurodesis. Although we believe that these variables were the most relevant clinically, we cannot exclude other influential factors, especially distinct genetic mutations. Exploring genotype-phenotype correlations were beyond the scope of this study. Finally, we did not look for correlations between lung function and the characteristics of cysts on HRCT. This issue deserves further study in a larger prospective cohort.

## Conclusion

The present study shows that, besides slightly increased RV and slightly reduced DLco, cystic lung disease in BHD does not affect lung function at baseline, and that no significant deterioration occurs over a follow-up period of 6 years, in sharp contrast with LAM. Accordingly, regular lung function follow-up does not seem necessary in BHD, unless lung involvement is extensive or the patient presents with respiratory symptoms or lung function impairment at baseline.

## Data Availability

The dataset used for the current study is available from the corresponding author on reasonable request.

## Abbreviations

BHD: Birt-Hogg-Dubé syndrome
DLco: Carbon monoxide transfer factor
DLco/V_A_: Carbon monoxide transfer coefficient
ERS: European Respiratory Society
FEV_1_: Forced expiratory volume in one second
FEV_1_/FVC: Forced expiratory volume in one second/forced vital capacity ratio
FVC: Forced vital capacity
GERMOP: Groupe d’Études et de Recherche sur les Maladies Orphelines Pulmonaires
HRCT: High-resolution computed tomography
LAM: Lymphangioleiomyomatosis
PaO_2_: Arterial oxygen partial pressure
RV: Residual volume
RV/TLC: Residual volume/total lung capacity ratio
SD: Standard deviation
TLC: Total lung capacity
